# Supplementary figures and images for: De novo identification of universal cell mechanics gene signatures
Source: eLife. 2025 Feb 17;12:RP87930. doi: 10.7554/eLife.87930 (PMC11832173; doi:10.7554/eLife.87930)

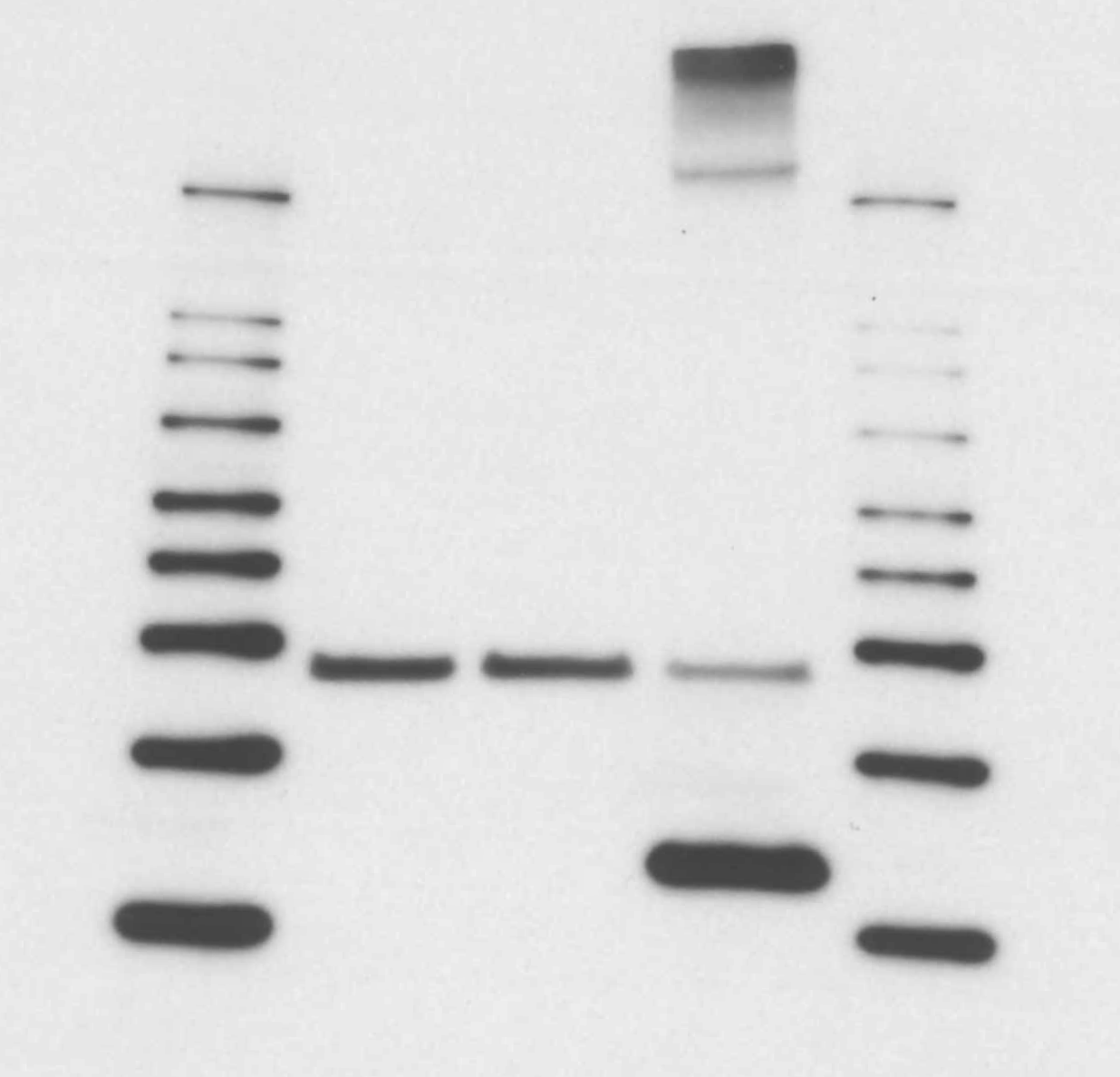

Supplement: Figure 5—source data 4. [file elife-87930-fig5-data4.zip › Fig5I-WB_CAV1OE_ECC4_replicate1.tif]

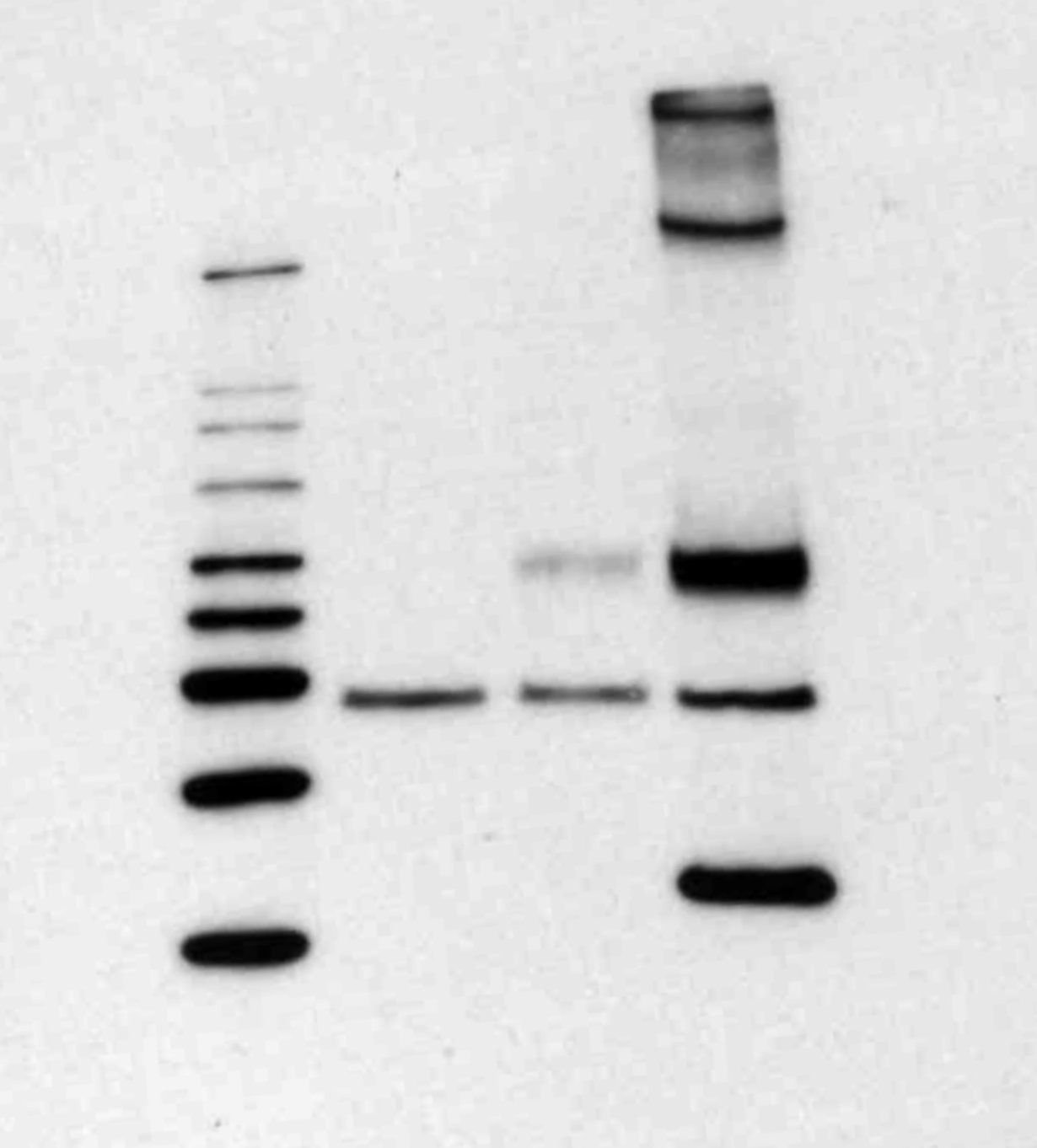

Supplement: Figure 5—source data 4. [file elife-87930-fig5-data4.zip › Fig5I-WB_CAV1OE_ECC4_replicate2.tif]

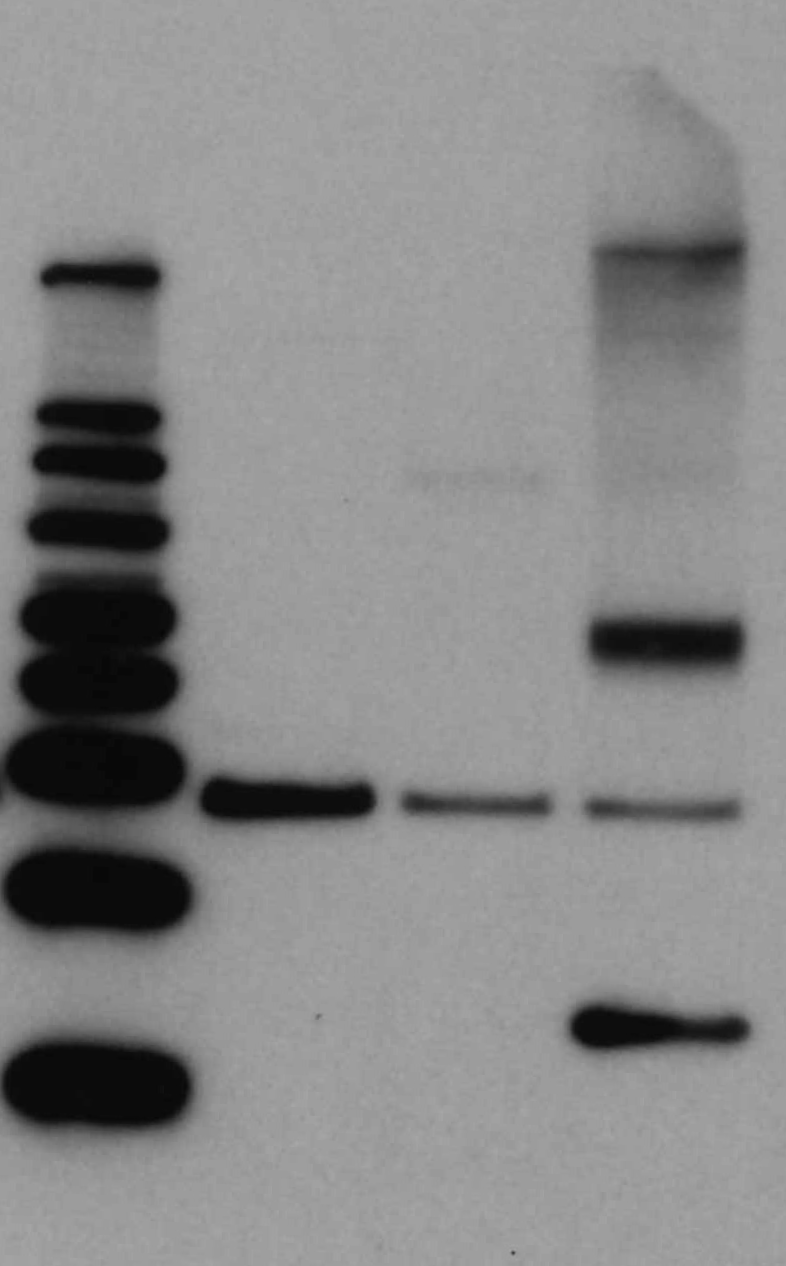

Supplement: Figure 5—source data 4. [file elife-87930-fig5-data4.zip › Fig5I-WB_CAV1OE_ECC4_replicate3.tif]

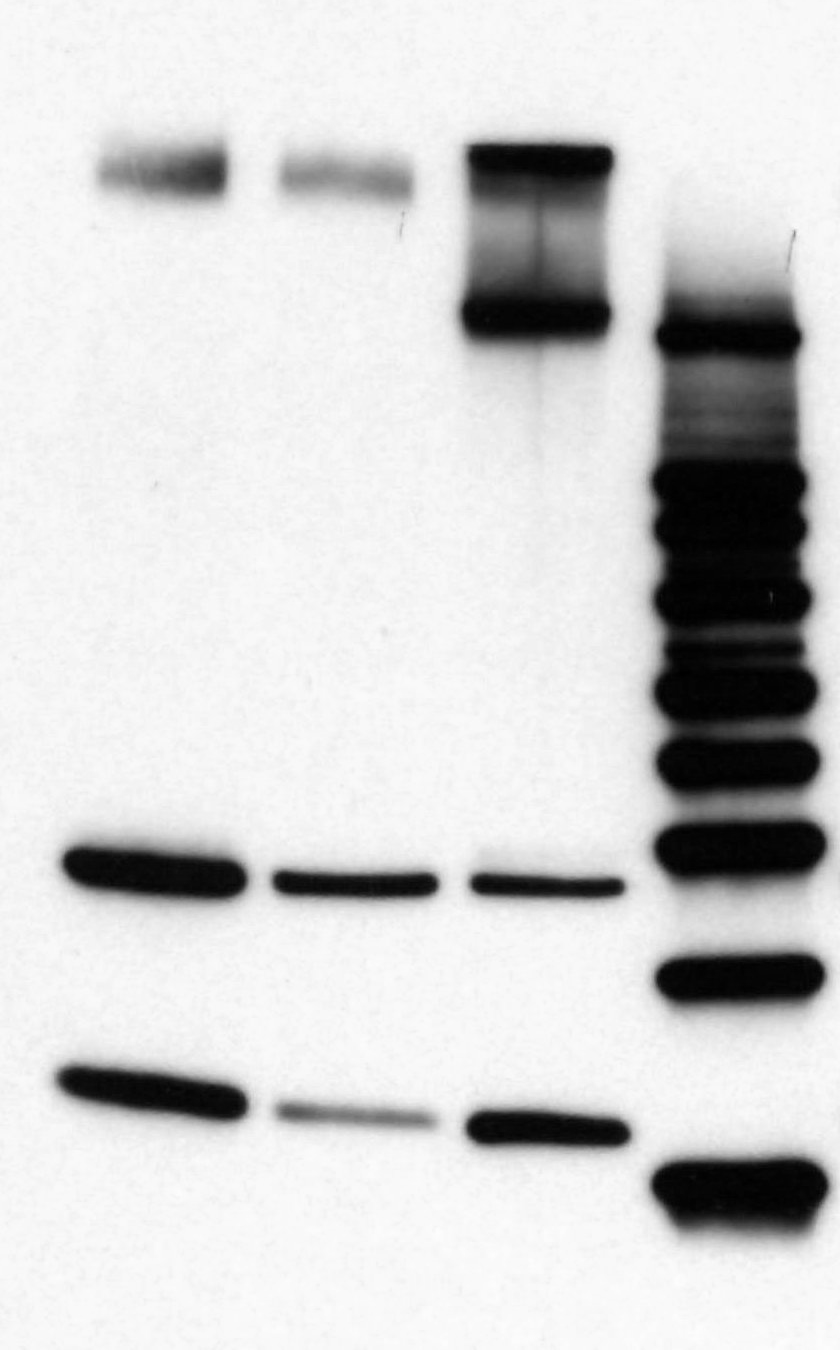

Supplement: Figure 5—source data 4. [file elife-87930-fig5-data4.zip › Fig5I-WB_CAV1OE_TGBC_replicate1.tif]

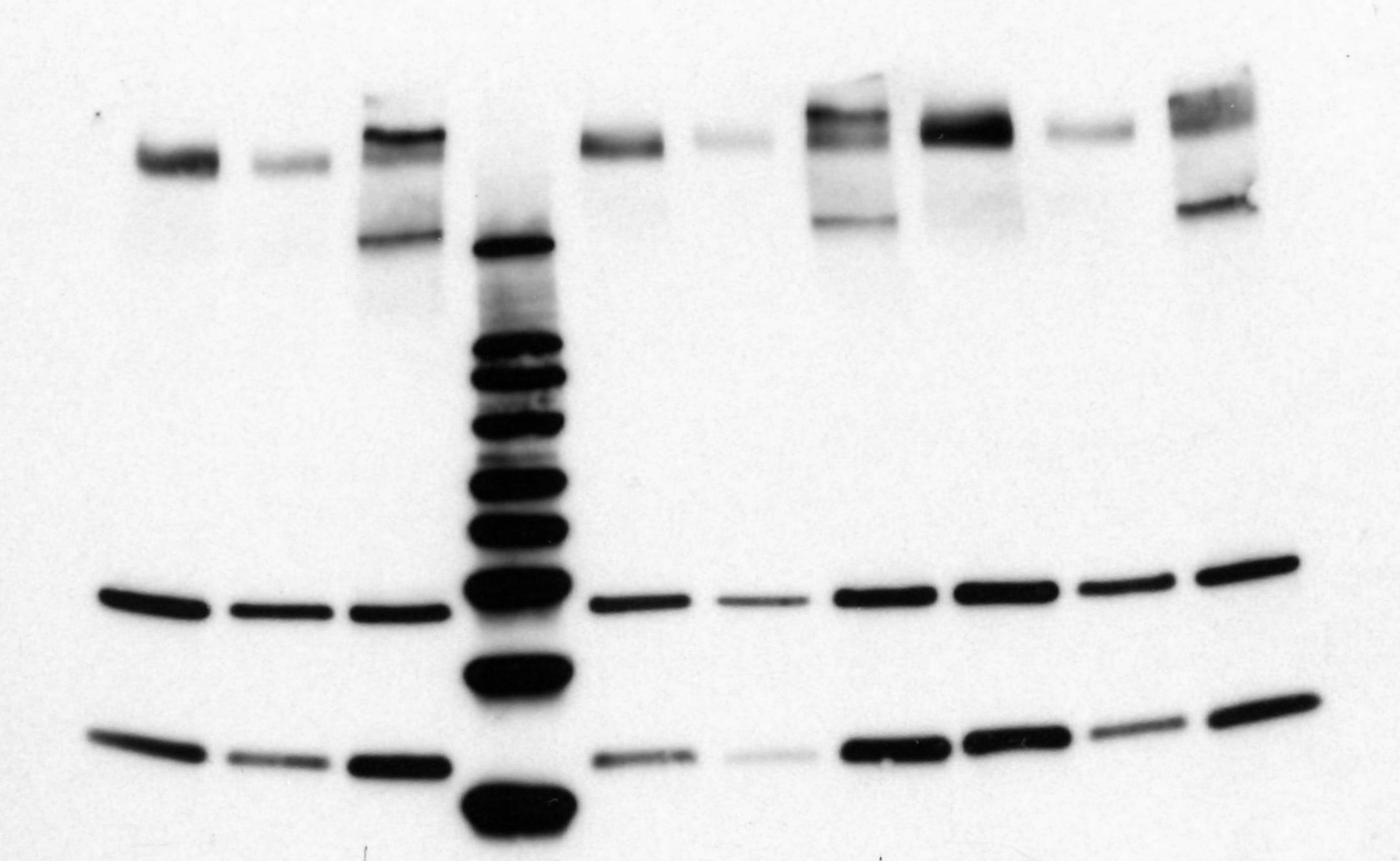

Supplement: Figure 5—source data 4. [file elife-87930-fig5-data4.zip › Fig5I-WB_CAV1OE_TGBC_replicate2-3.tif]

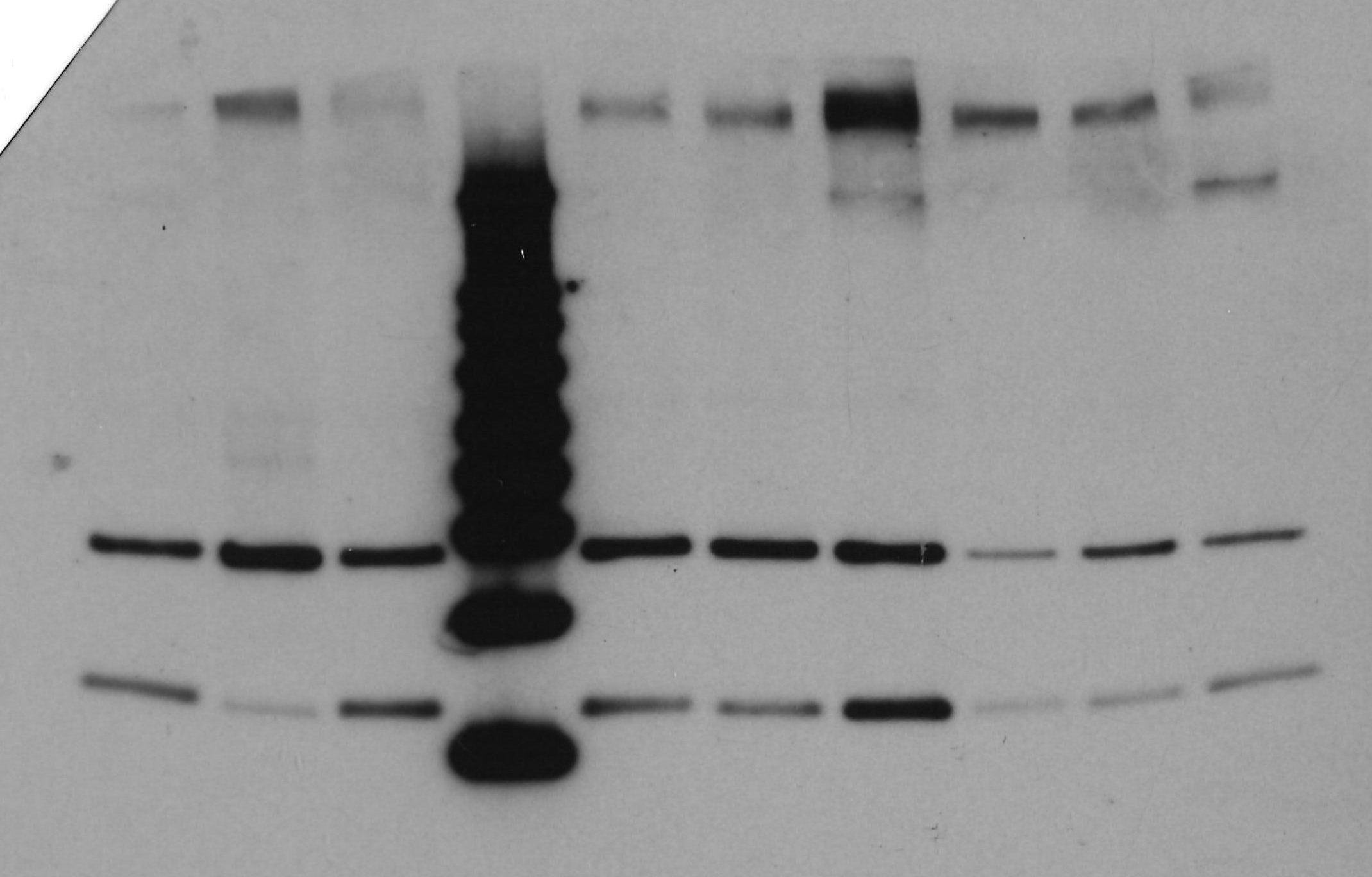

Supplement: Figure 5—source data 4. [file elife-87930-fig5-data4.zip › Fig5I-WB_CAV1OE_TGBC_replicate4-6.tif]

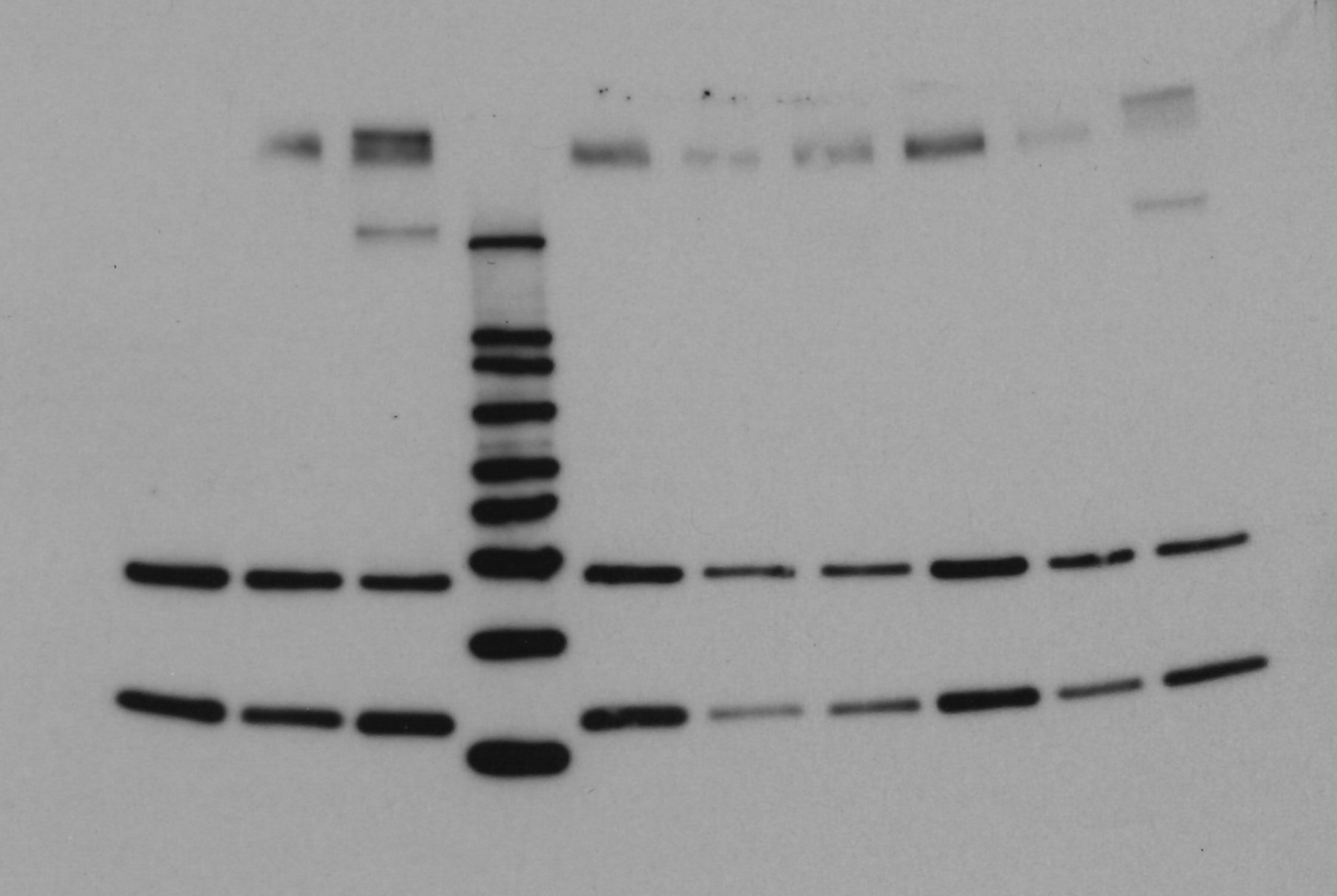

Supplement: Figure 5—source data 4. [file elife-87930-fig5-data4.zip › Fig5I-WB_CAV1OE_TGBC_replicate7-9.tif]

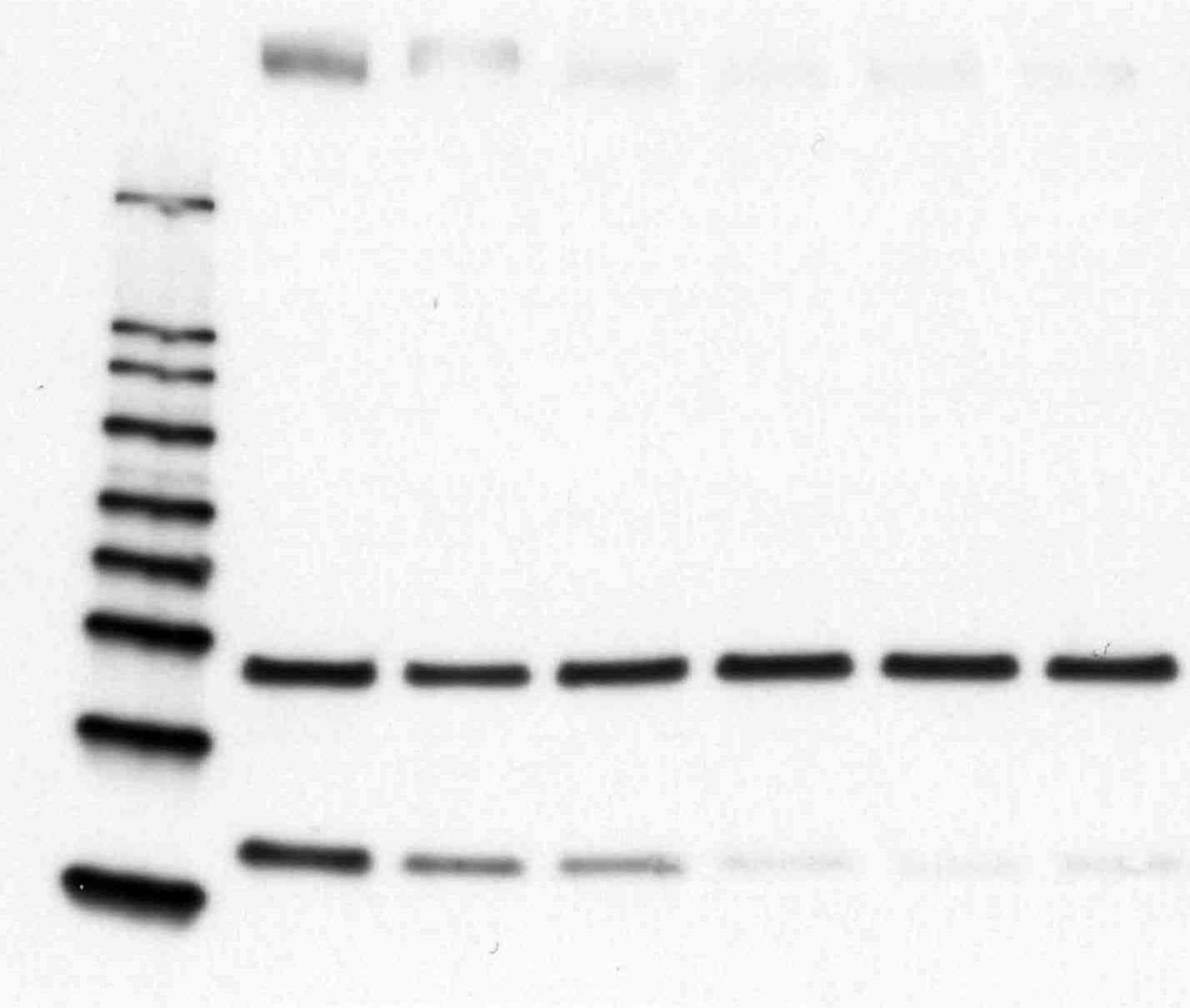

Supplement: Figure 5—source data 4. [file elife-87930-fig5-data4.zip › Fig5E-WB_TGBC-CAV1KD_esiRNA_replicate4.tif]

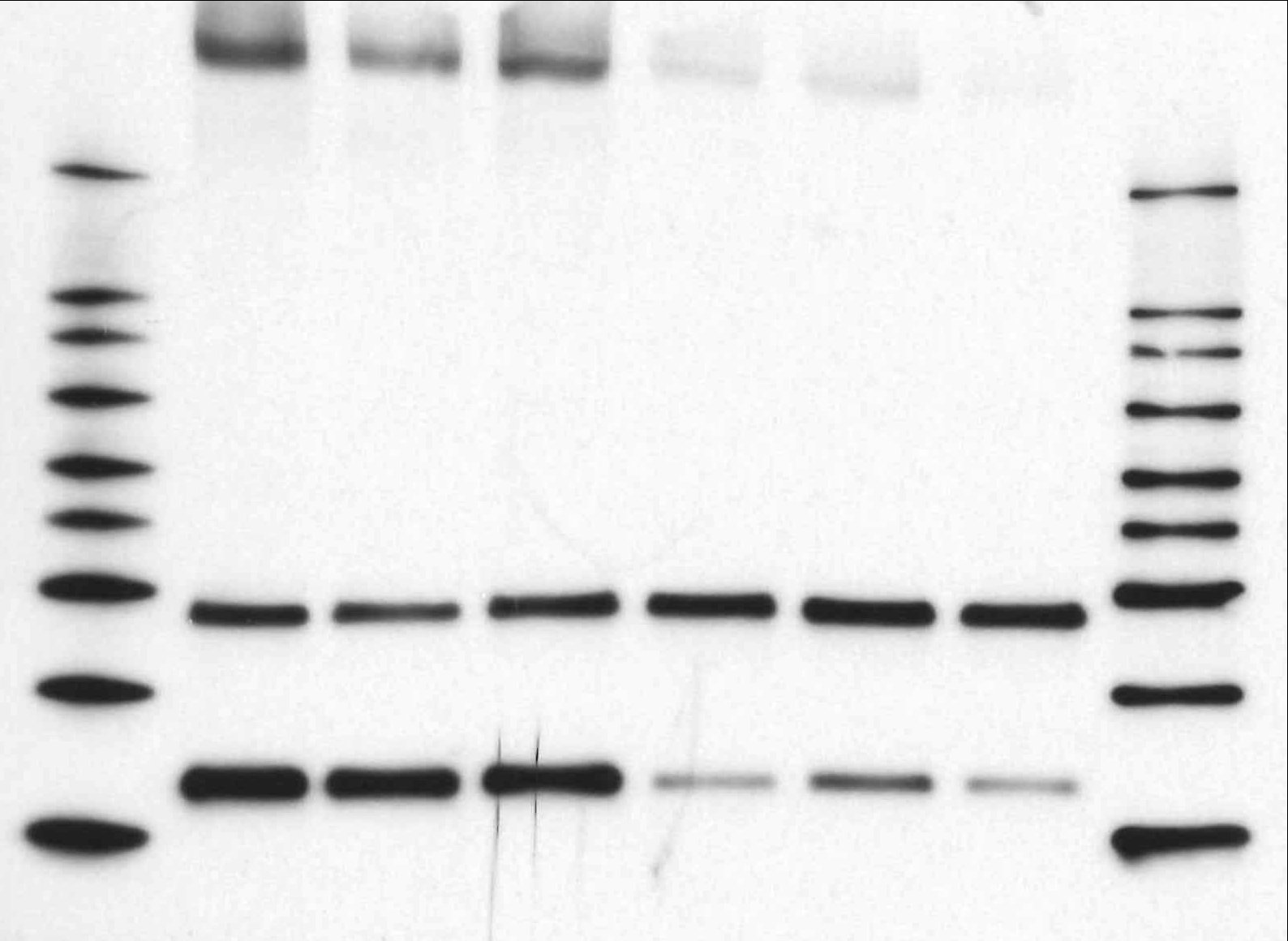

Supplement: Figure 5—source data 4. [file elife-87930-fig5-data4.zip › Fig5E-WB_TGBC-CAV1KD_esiRNA_replicate3.tif]

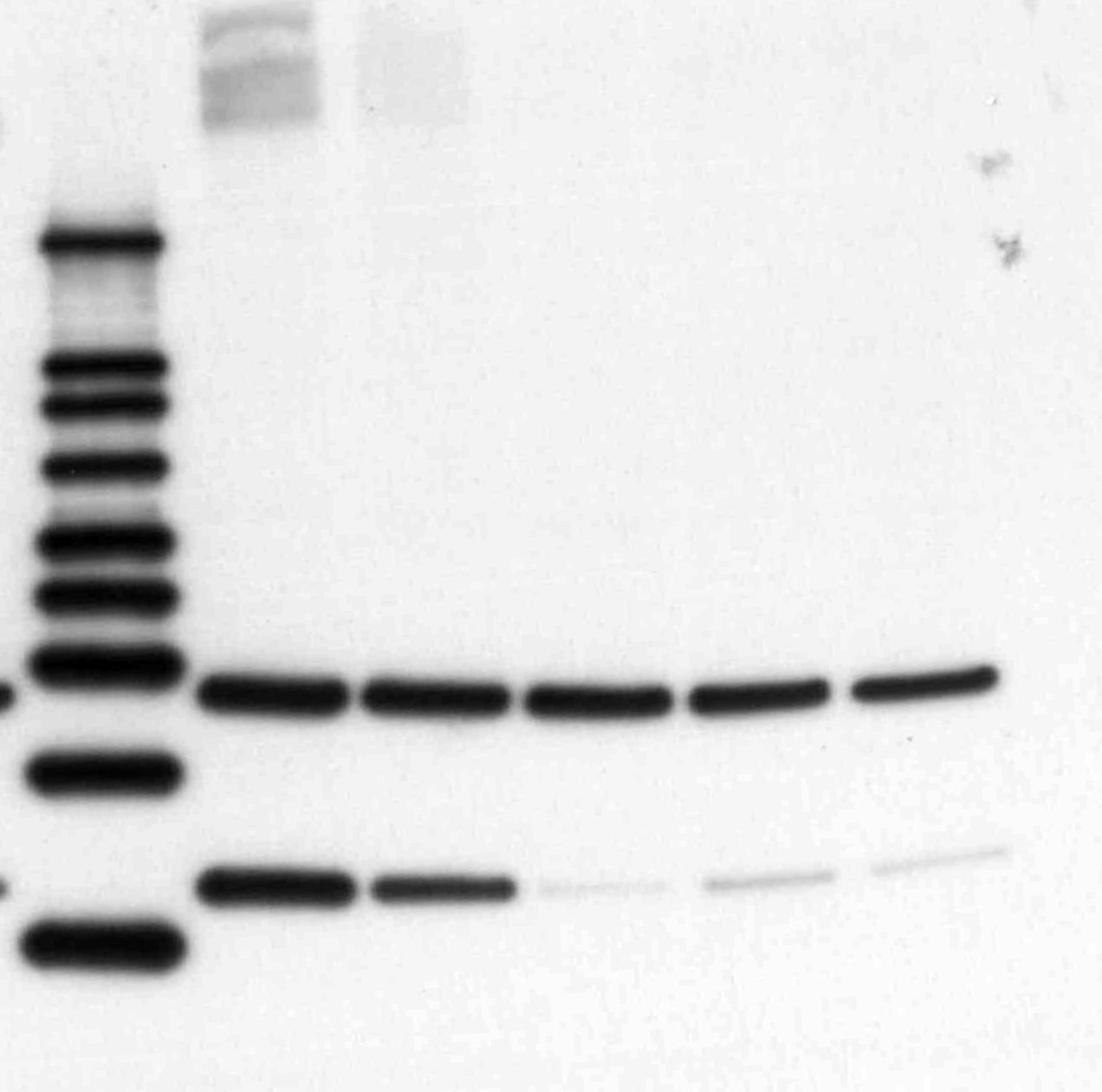

Supplement: Figure 5—source data 4. [file elife-87930-fig5-data4.zip › Fig5E-WB_TGBC-CAV1KD_esiRNA_replicate2.tif]

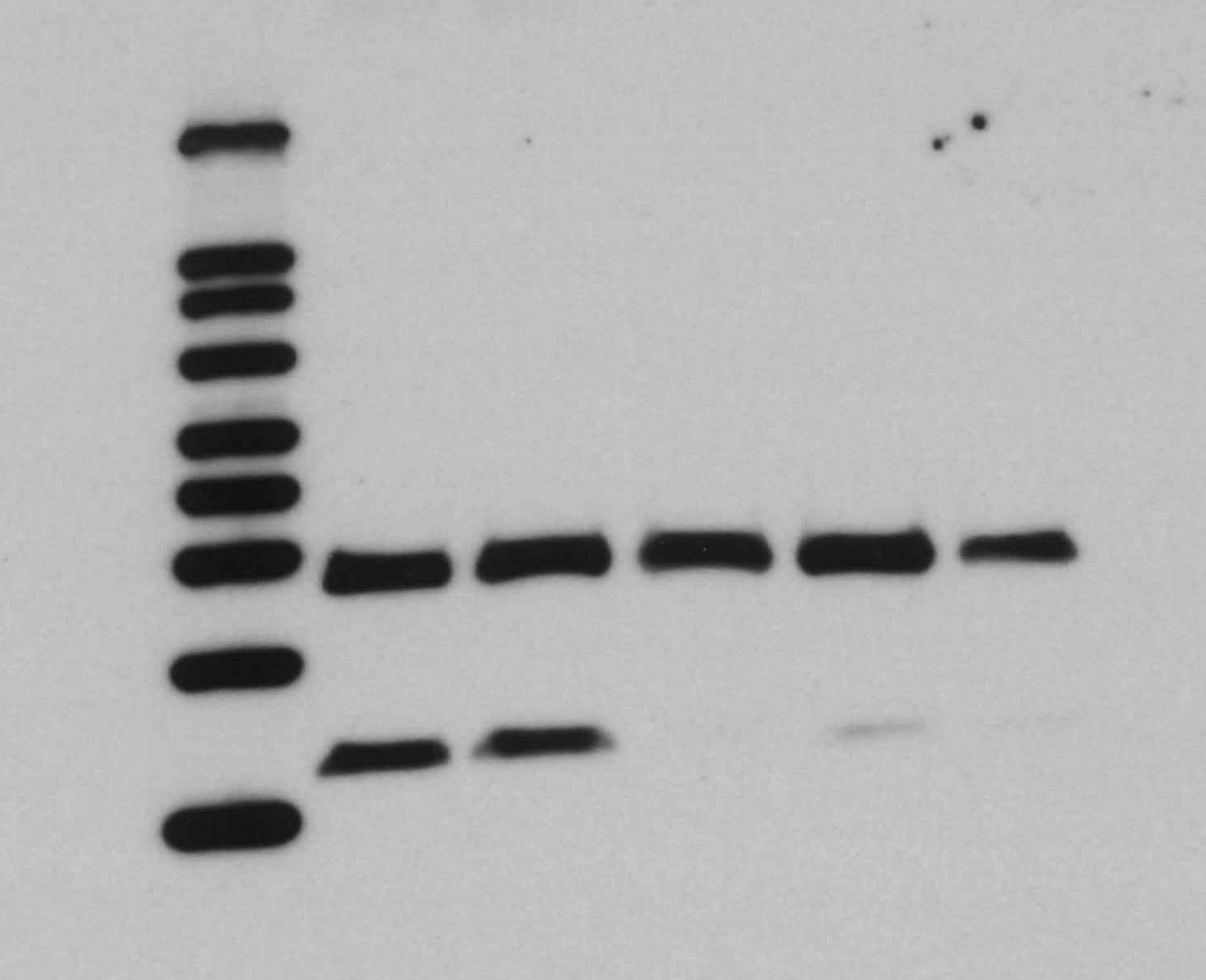

Supplement: Figure 5—source data 4. [file elife-87930-fig5-data4.zip › Fig5E-WB_TGBC-CAV1KD_esiRNA_replicate1.tif]

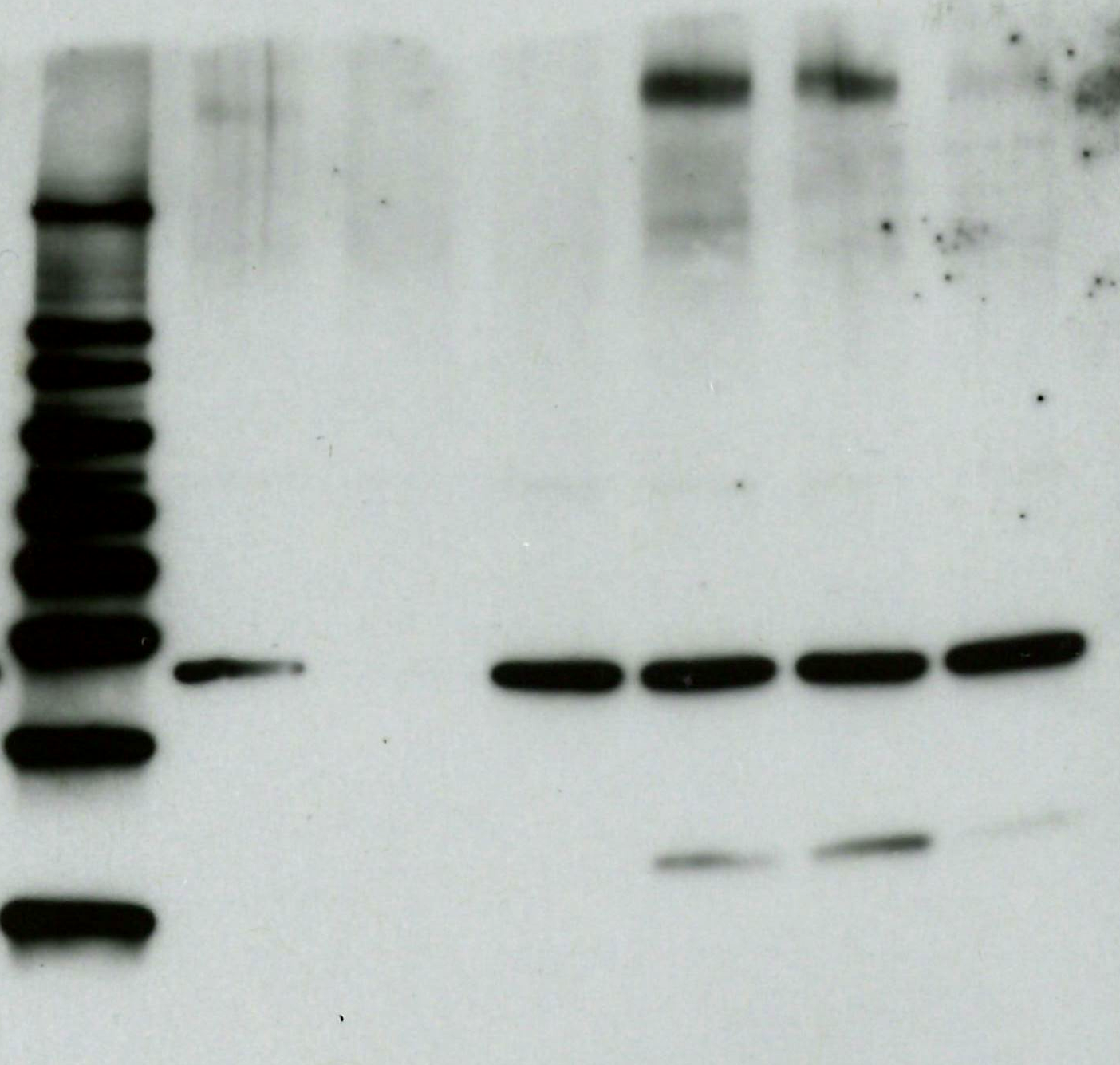

Supplement: Figure 5—source data 4. [file elife-87930-fig5-data4.zip › Fig5E-WB_TGBC-CAV1KD_ONTarget_replicate4.tif]

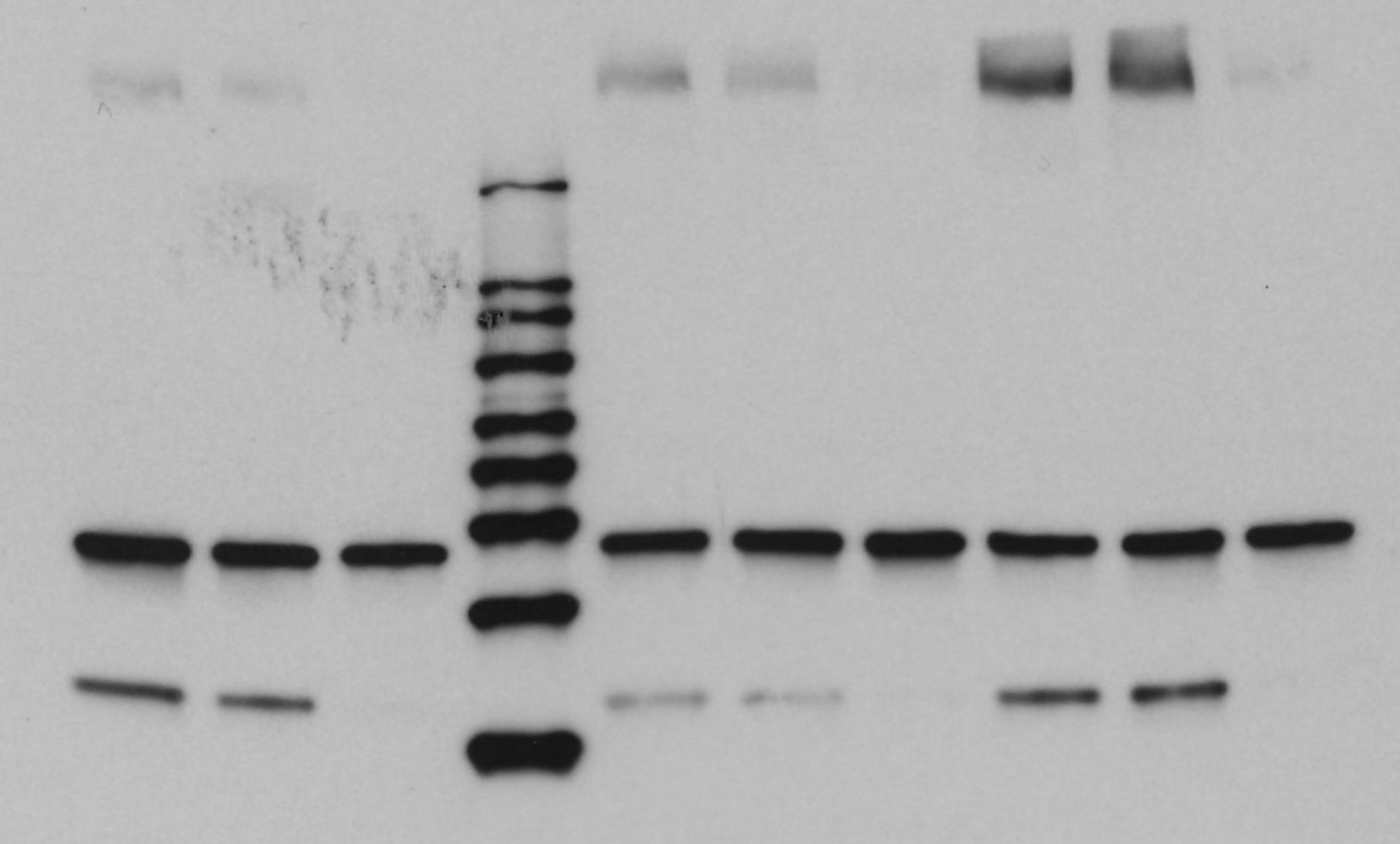

Supplement: Figure 5—source data 4. [file elife-87930-fig5-data4.zip › Fig5E-WB_TGBC-CAV1KD_ONTarget_replicate1-3.tif]

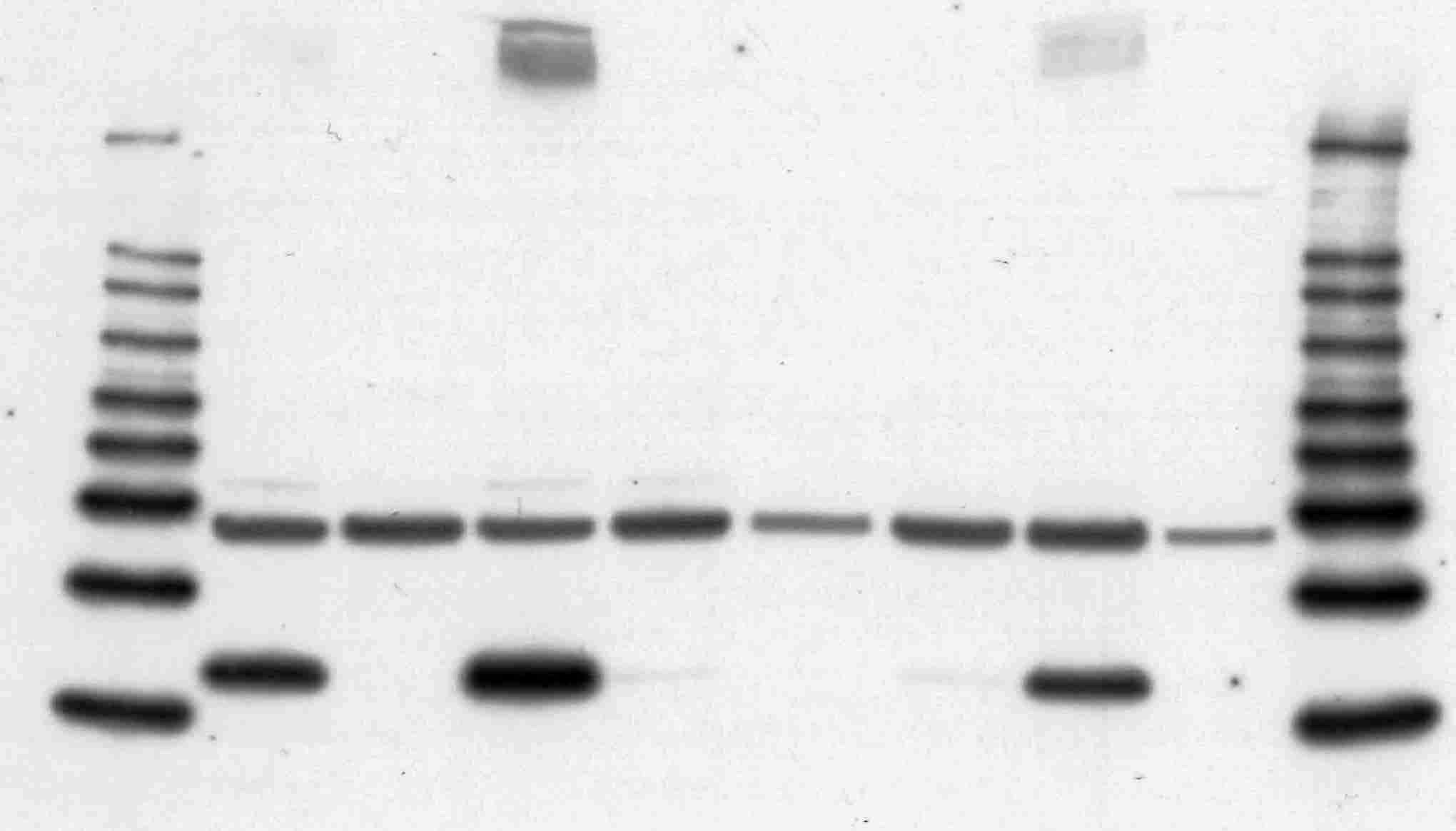

Supplement: Figure 5—source data 4. [file elife-87930-fig5-data4.zip › Fig5A-WB_Carcinoma_CAV1_replicate1.tif]

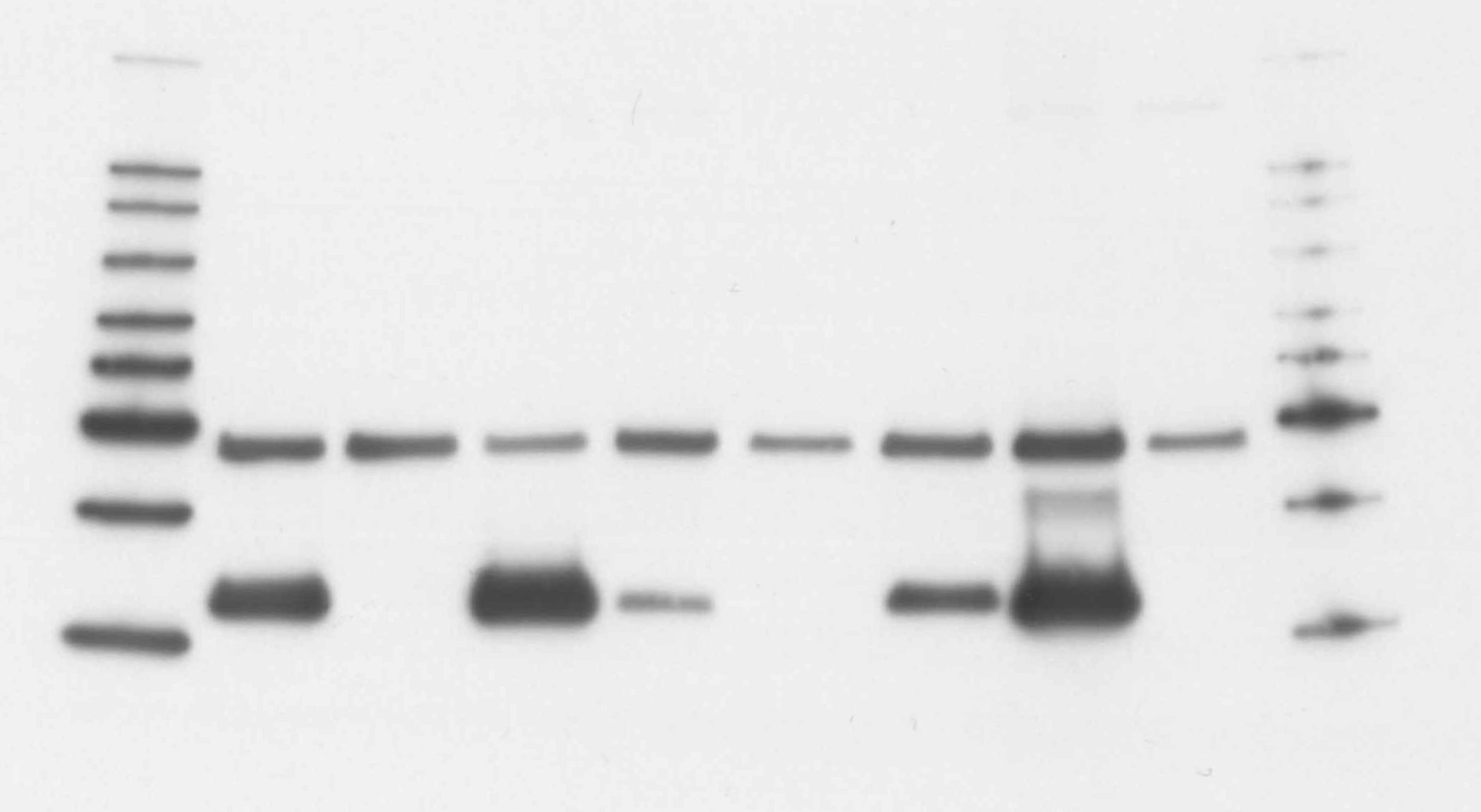

Supplement: Figure 5—source data 4. [file elife-87930-fig5-data4.zip › Fig5A-WB_Carcinoma_CAV1_replicate2.tif]

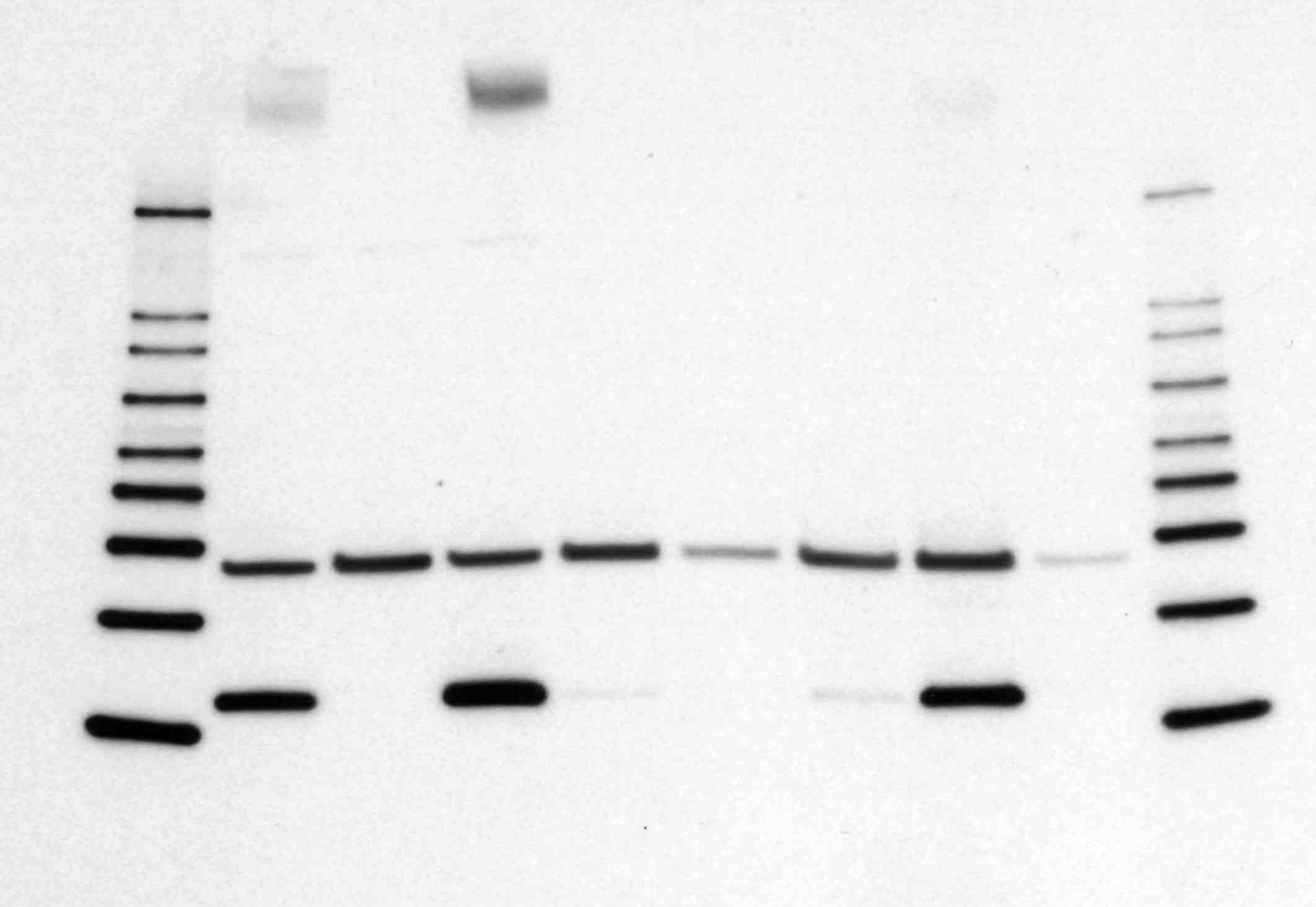

Supplement: Figure 5—source data 4. [file elife-87930-fig5-data4.zip › Fig5A-WB_Carcinoma_CAV1_replicate3.tif]

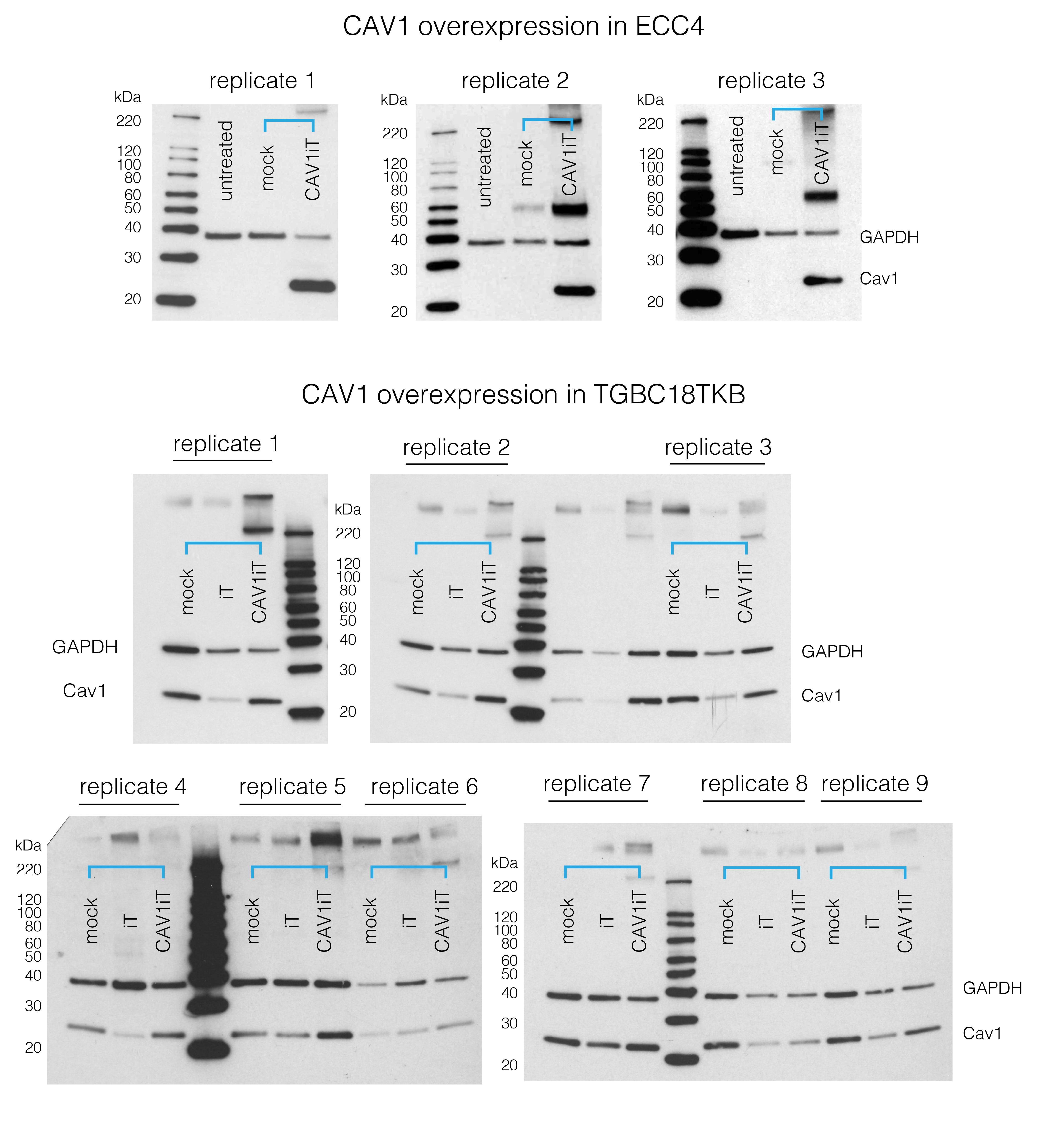

Supplement: Figure 5—source data 5. [file elife-87930-fig5-data5.zip › Fig5I-WB_CAV1OE_All_overview.png]

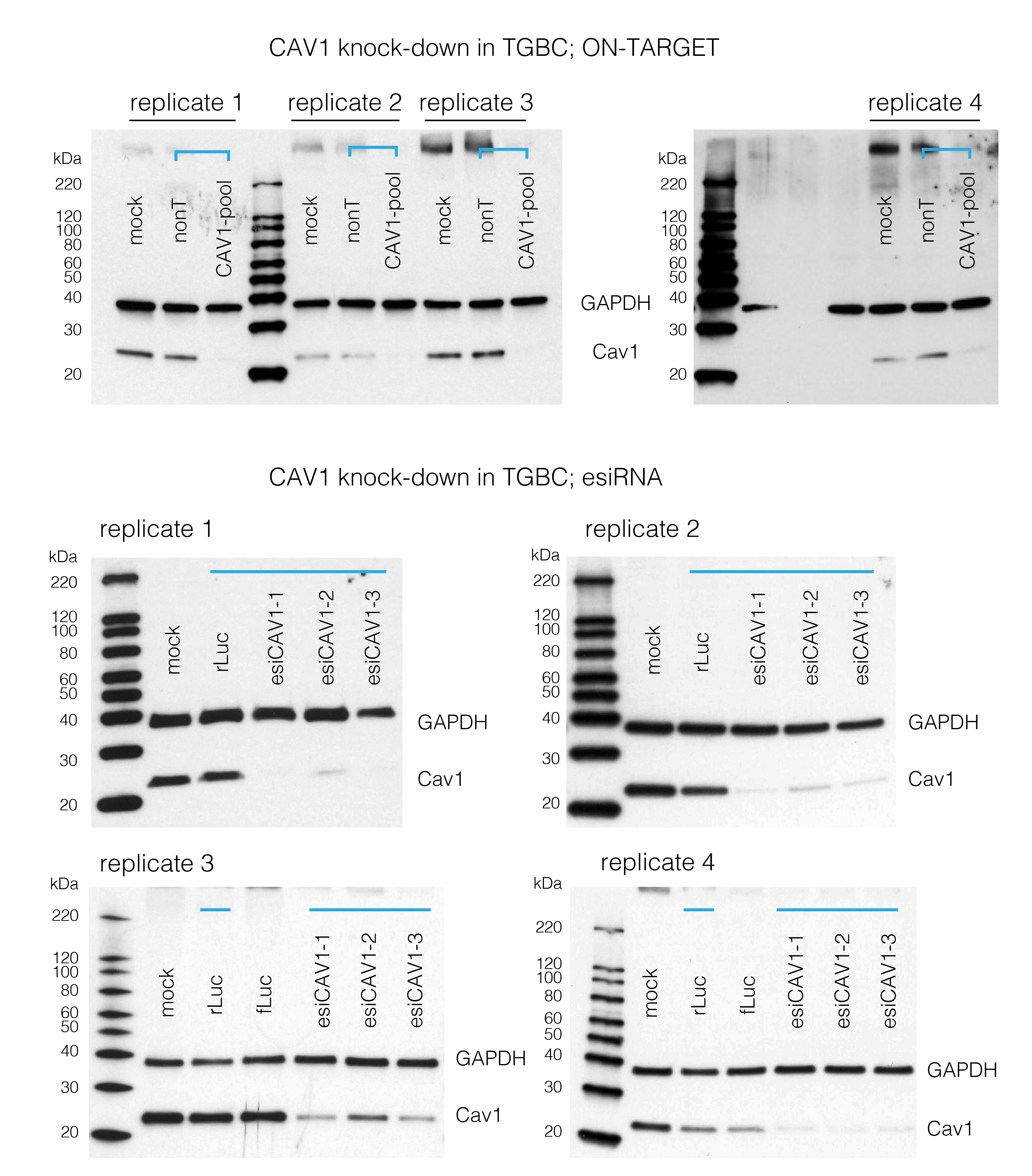

Supplement: Figure 5—source data 5. [file elife-87930-fig5-data5.zip › Fig5E-WB_TGBC-CAV1KD_overview.png]

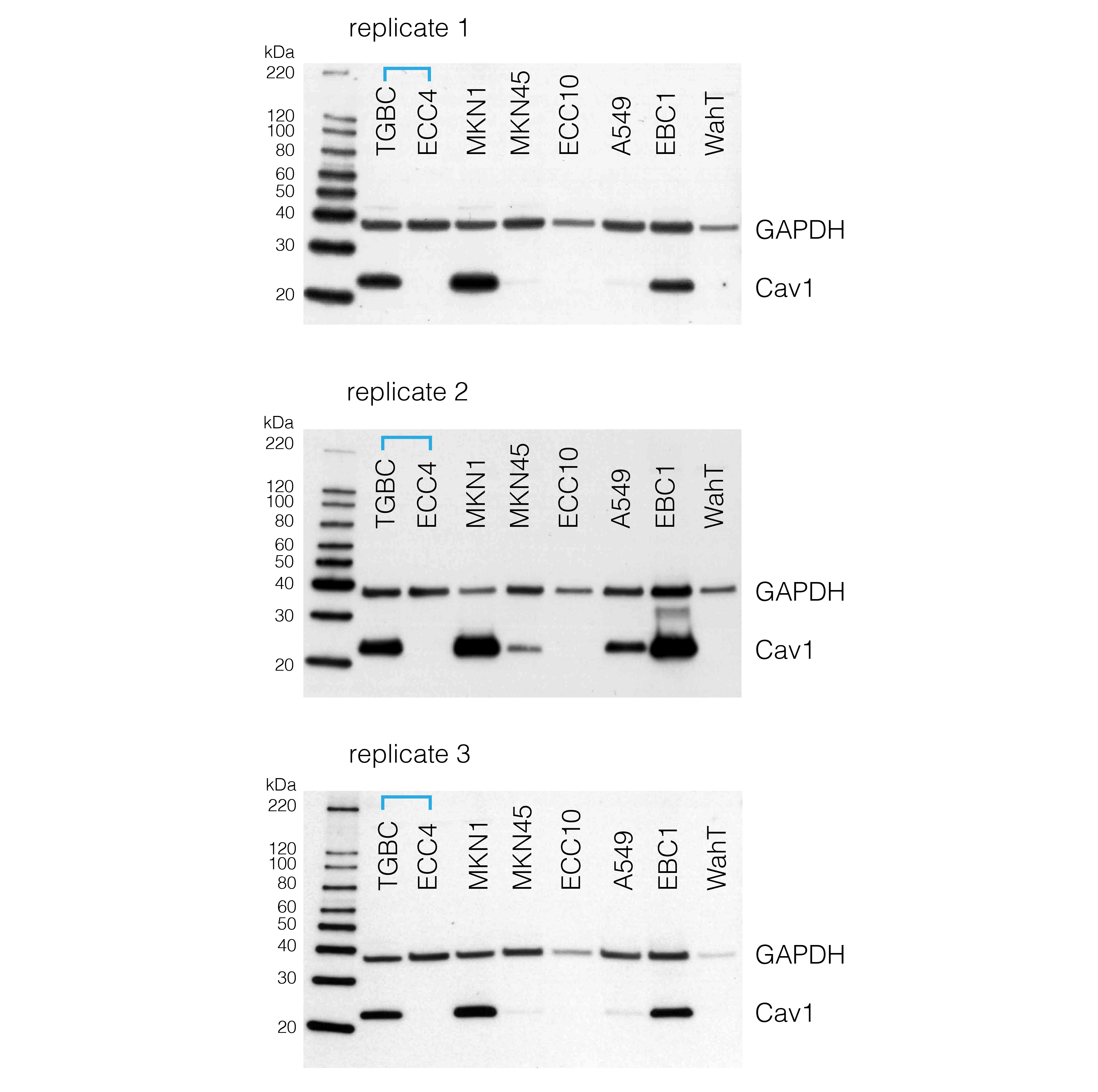

Supplement: Figure 5—source data 5. [file elife-87930-fig5-data5.zip › Fig5A-WB_Carcinoma_CAV1_overview.png]

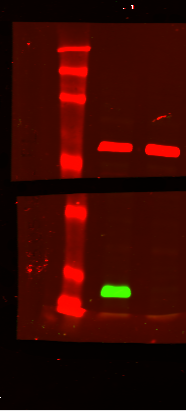

Supplement: Figure 5—figure supplement 1—source data 1. [file elife-87930-fig5-figsupp1-data1.zip › Fig5ΓÇöfigureSupplement1A-WB_MEFs-CAV1KO.tif]

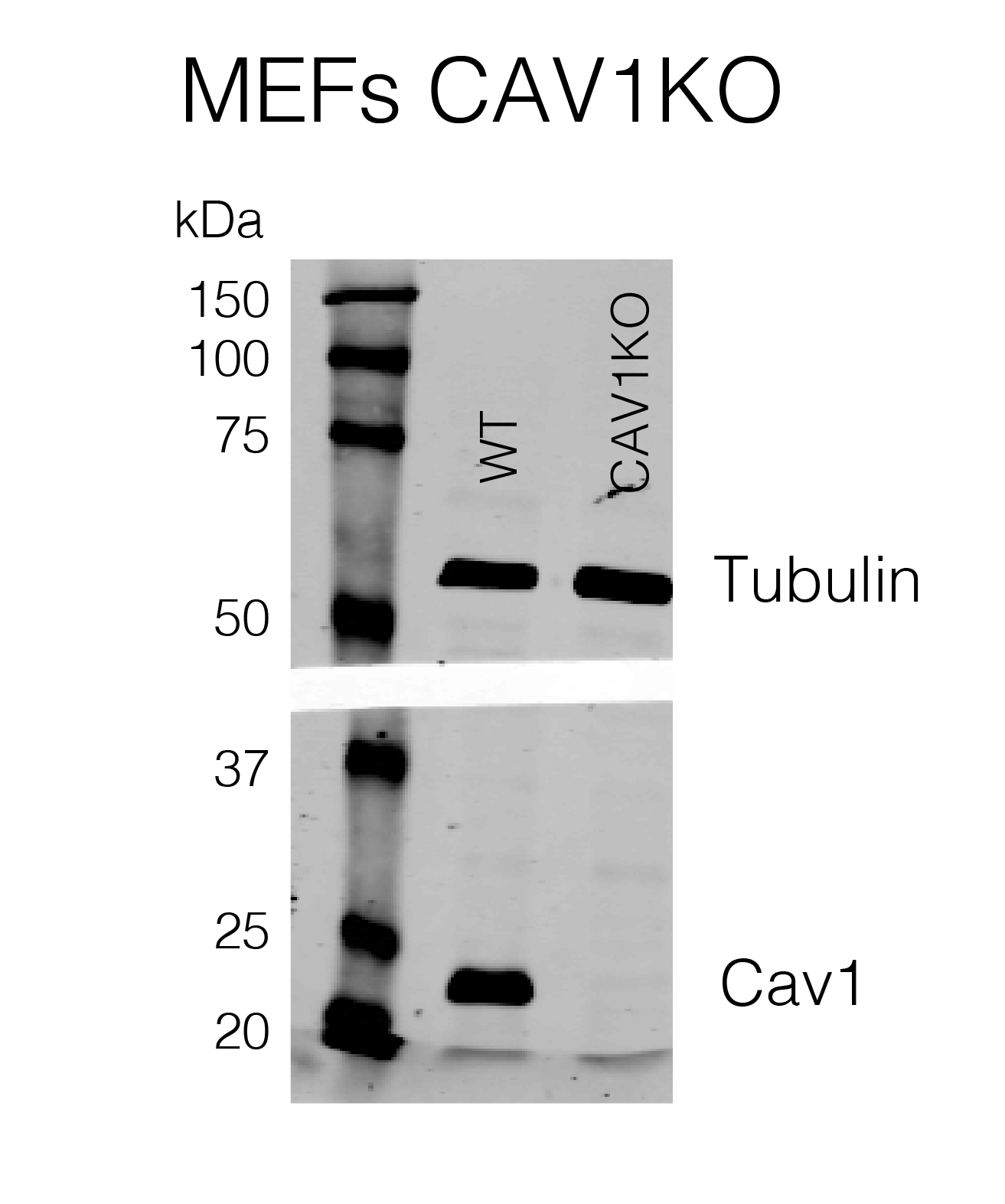

Supplement: Figure 5—figure supplement 1—source data 2. [file elife-87930-fig5-figsupp1-data2.zip › Fig5ΓÇöfigureSupplement1A-WB_MEFs-CAV1KO_overview.png]

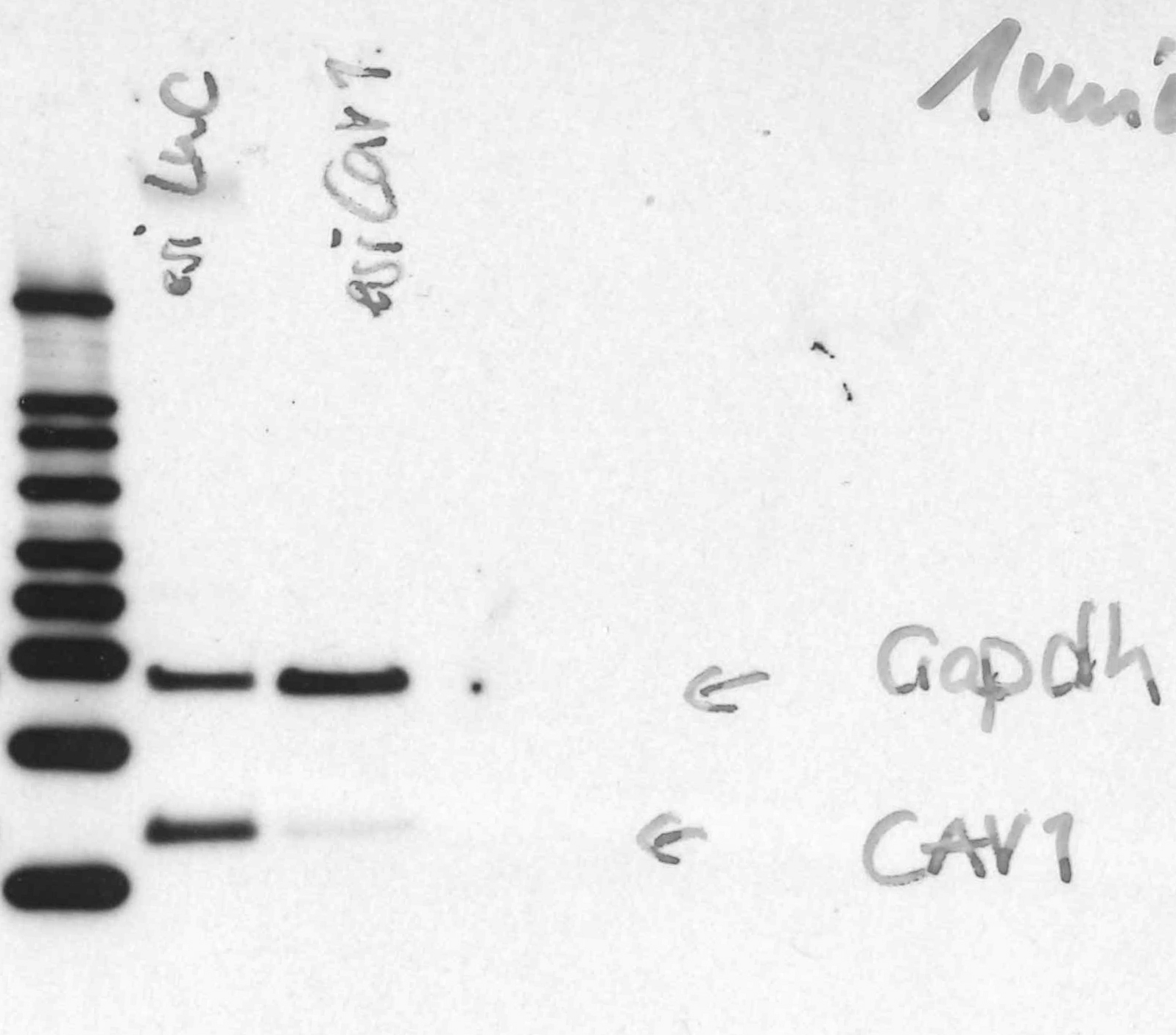

Supplement: Figure 6—source data 3. [file elife-87930-fig6-data3.zip › Fig6DE_MCF10A-ER-Src_CAV1_KDrep1.tif]

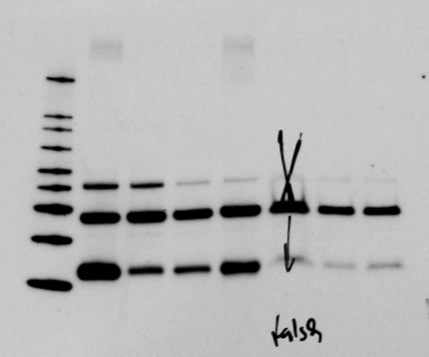

Supplement: Figure 6—source data 3. [file elife-87930-fig6-data3.zip › Fig6DE_MCF10A-ER-Src_CAV1_KDrep2_OErep1n2.tif]

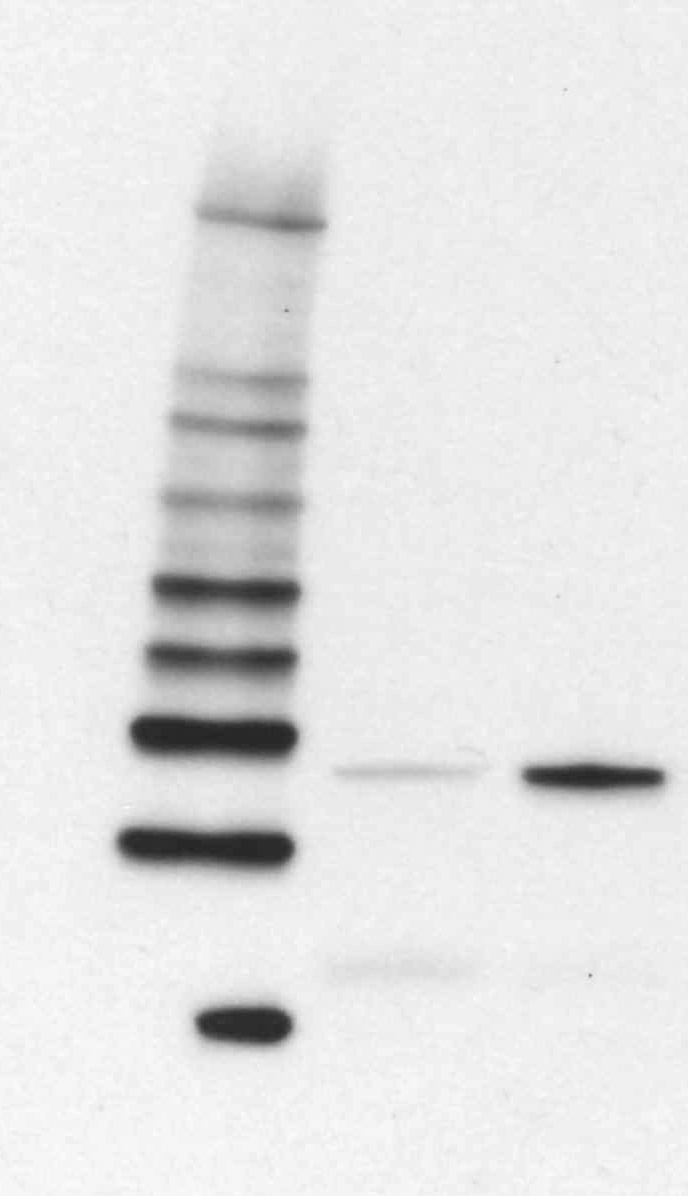

Supplement: Figure 6—source data 3. [file elife-87930-fig6-data3.zip › Fig6DE_MCF10A-ER-Src_CAV1_KDrep3.tif]

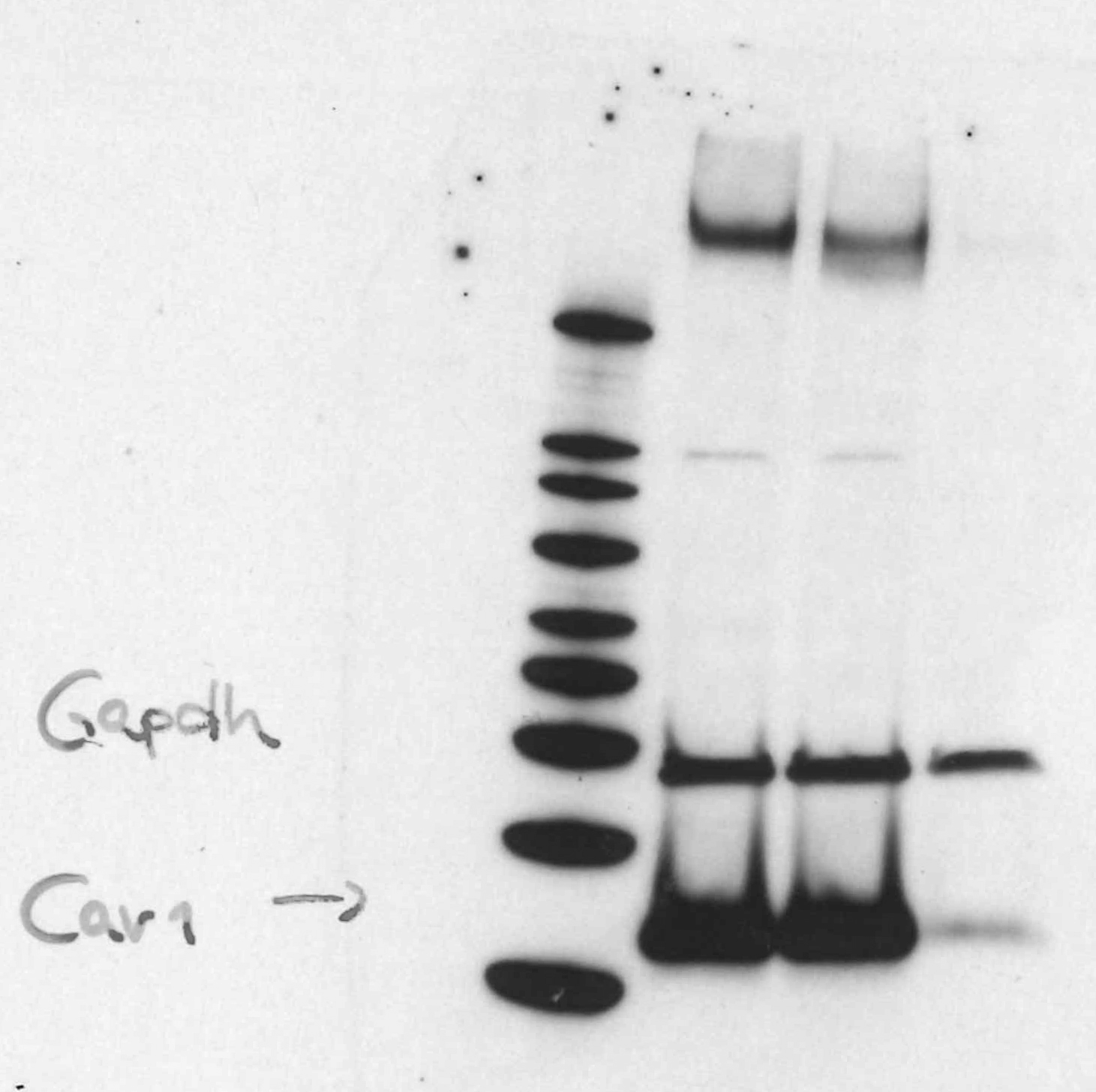

Supplement: Figure 6—source data 3. [file elife-87930-fig6-data3.zip › Fig6B_MCF10A-ER-Src_TAMind_48h_72h_rep1.tif]

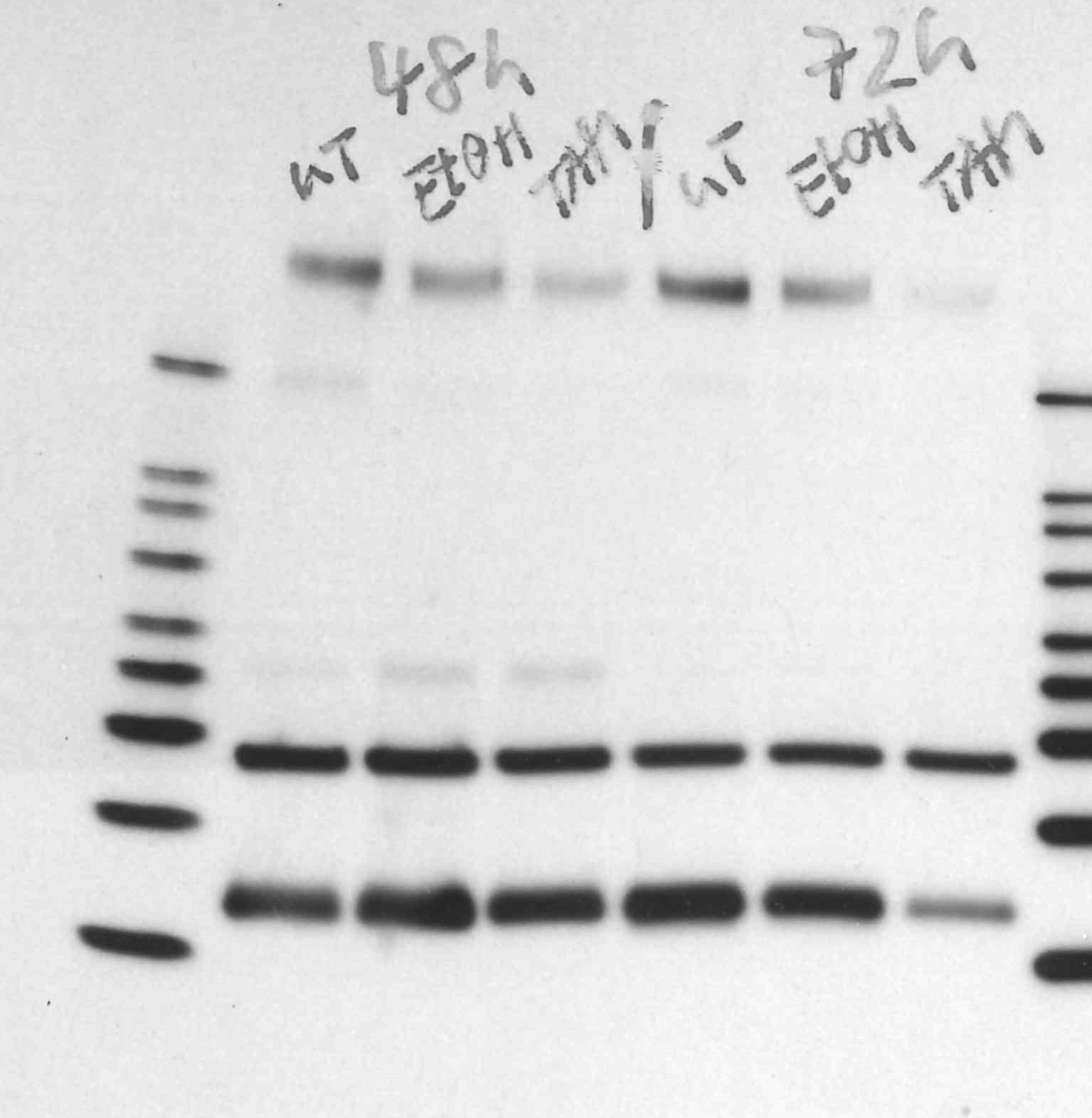

Supplement: Figure 6—source data 3. [file elife-87930-fig6-data3.zip › Fig6B_MCF10A-ER-Src_TAMind_48h_72h_rep2.tif]

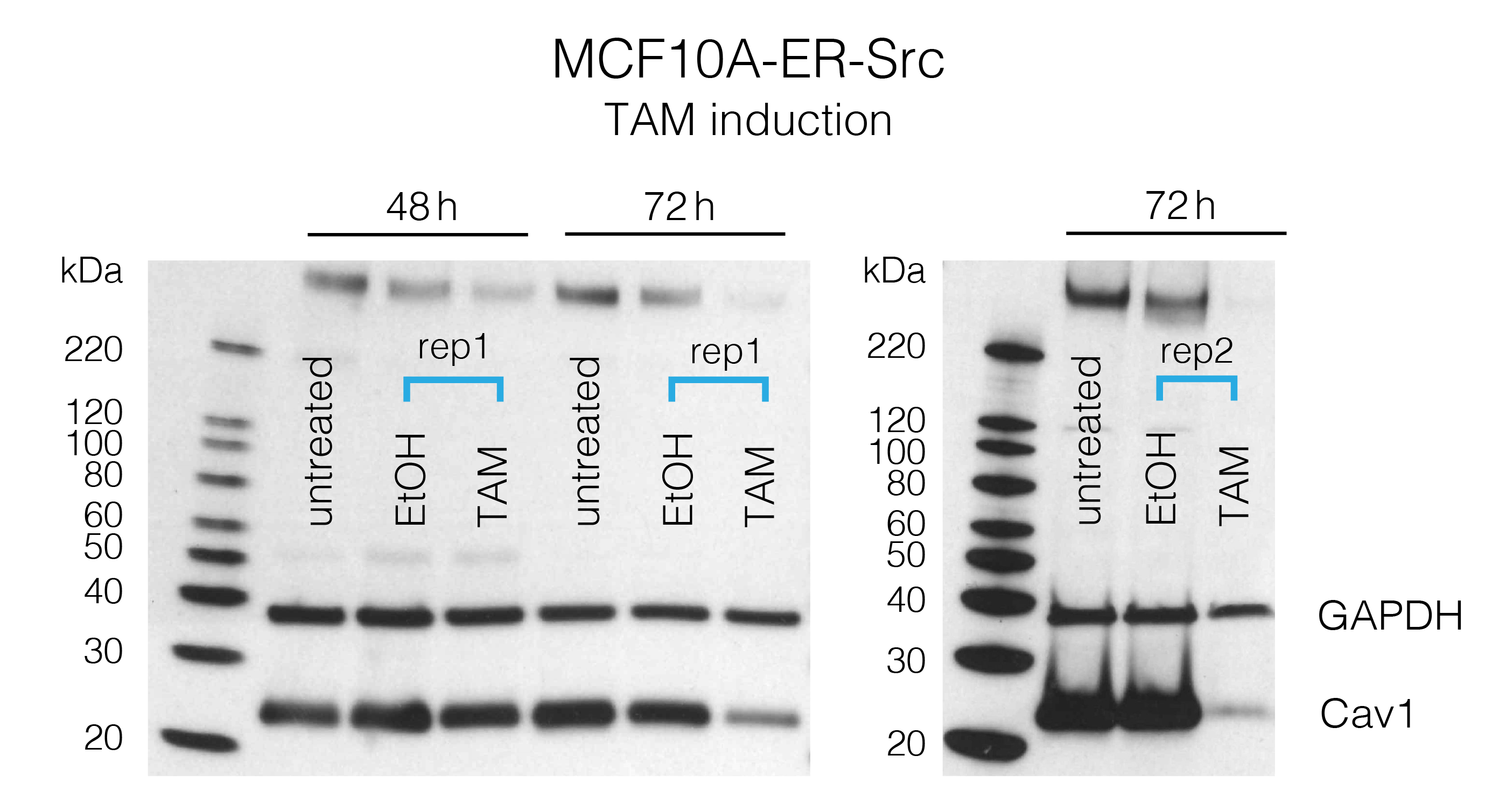

Supplement: Figure 6—source data 4. [file elife-87930-fig6-data4.zip › Fig6B_MCF10A-ER-Src_TAMind_overview.png]

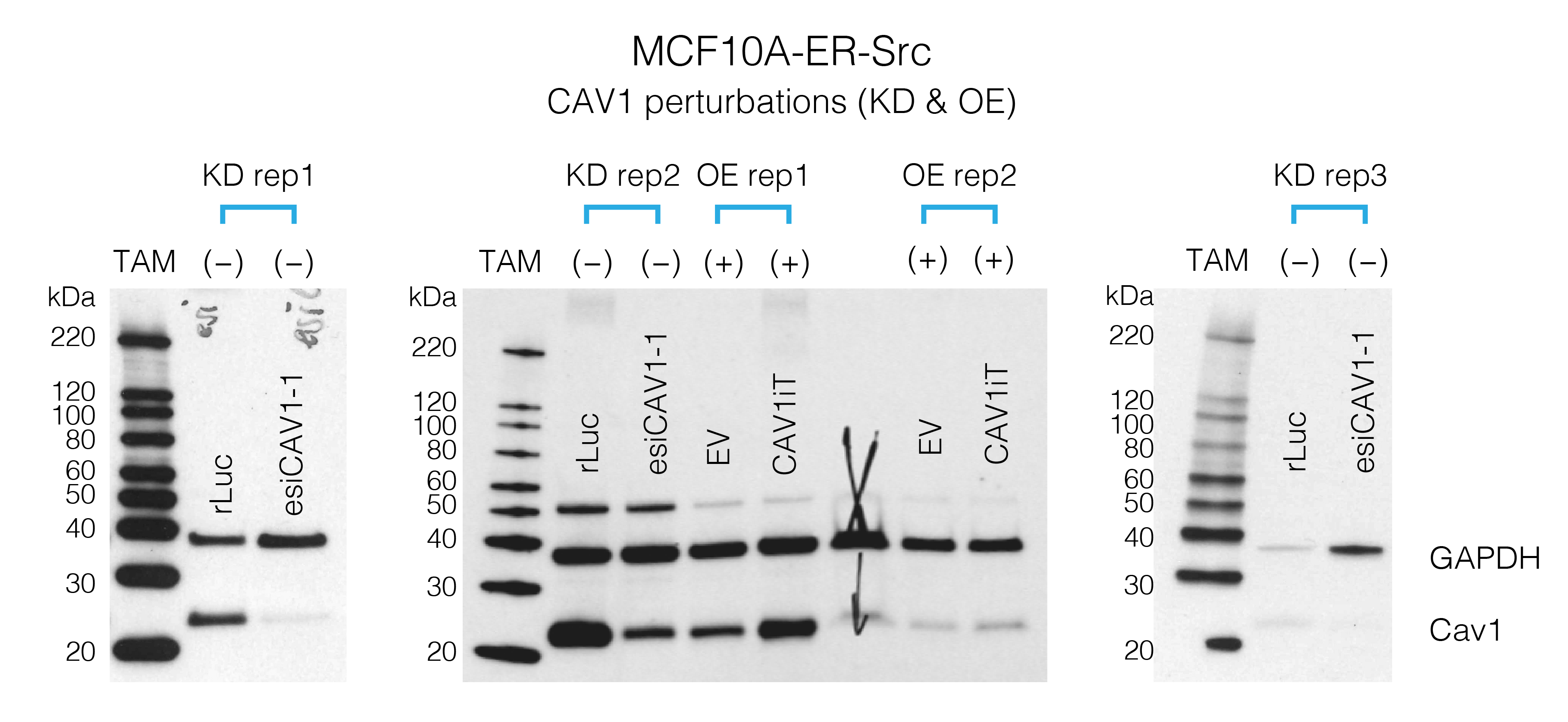

Supplement: Figure 6—source data 4. [file elife-87930-fig6-data4.zip › Fig6DE_MCF10A-ER-Src_CAV1perturb_overview.png]
