# Supplementary material for: De novo identification of universal cell mechanics gene signatures
Source: eLife. 2025 Feb 17;12:RP87930. doi: 10.7554/eLife.87930 (PMC11832173; doi:10.7554/eLife.87930)
Supplement: Supplementary file 1. [file elife-87930-supp1.docx]

**Supplementary File 1**

**Operation parameters of the three methods used for characterizing the mechanical properties of cells.**

The table below summarizes the main parameters characteristic for the three methods applied for characterizing the mechanical properties of cells in this study: AFM indentation, AFM microrheology, and RT-DC. For AFM indentation, the setpoint of 2 nN leads to a resulting deformation on the order of 1 μm; for AFM microrheology, the deformation corresponds to the magnitude of the cantilever oscillations which was set to 10 nm. The corresponding strains are 7% for AFM indentation and 0.07% for AFM microrheology. For RTDC, a mean absolute strain on the order of 17% was reported (72), this corresponds to ca 2.5 μm deformation. For the above estimation, the cell diameter was assumed to be 15 μm (ECC4 and TGBC cells have a cross-section area within 100–300 μm^2^ (see Figure 2—figure supplement 1B), corresponding to 11.3–19.5 μm diameter). For RT-DC, the probing frequency was deduced based on the time it takes to pass a 300-μm long square channel with an average velocity based on a total flow rate of 0.16 μl s^−1^ and 30 μm channel width (a setting most frequently used in this study, see Table S5), For AFM microrheology, the frequency is equivalent to the oscillation frequency; and for AFM indentation, it is deduced from the extension speed of 5 μm s^−1^ and an average deformation of 1 μm.

|  | **AFM indentation** | **AFM microrheology** | **RT-DC** |
| --- | --- | --- | --- |
| **cell state** | adherent | adherent | suspended |
| **probing lengthscale** | local (5-μm bead) | local (5-μm bead) | whole-cell |
| **frequency (probing timescale)** | 5 Hz (200 ms) | 3 Hz (300 ms) – 200 Hz (5 ms) | ~ 600 Hz (1.67 ms) |
| **induced deformation** | 1 μm | 10 nm | 2.5 μm |
| **strain** | 7 % | 0.07 % | 17 % |
| **deformation rate** | 5 μm s^−1^ | 0.03–2 μm s^−1^ | 1.5 mm s^−1^ |
| **strain rate** | 0.35 Hz | 0.002–0.140 Hz | 102 Hz |
