# Supplementary material for: De novo identification of universal cell mechanics gene signatures
Source: eLife. 2025 Feb 17;12:RP87930. doi: 10.7554/eLife.87930 (PMC11832173; doi:10.7554/eLife.87930)
Supplement: Supplementary file 5. [file elife-87930-supp5.docx]

**Supplementary File 5**

**Sequences of esiRNAs used for CAV1 knock-down experiments**

>HU-03125-1 (414 bp)

GAGCTGAGCGAGAAGCAAGTGTACGACGCGCACACCAAGGAGATCGACCT
GGTCAACCGCGACCCTAAACACCTCAACGATGACGTGGTCAAGATTGACT
TTGAAGATGTGATTGCAGAACCAGAAGGGACACACAGTTTTGACGGCATT
TGGAAGGCCAGCTTCACCACCTTCACTGTGACGAAATACTGGTTTTACCG
CTTGCTGTCTGCCCTCTTTGGCATCCCGATGGCACTCATCTGGGGCATTT
ACTTCGCCATTCTCTCTTTCCTGCACATCTGGGCAGTTGTACCATGCATT
AAGAGCTTCCTGATTGAGATTCAGTGCATCAGCCGTGTCTATTCCATCTA
CGTCCACACCGTCTGTGACCCACTCTTTGAAGCTGTTGGGAAAATATTCA
GCAATGTCCGCATC

>HU-03125-2 (325 bp)

CCAAAATGTTGGTCATTTTATGTTAAGGGAAGAATTCCAGGGTATGGCCA
TGGAGTGTACAAGTATGTGGGCAGATTTTCAGCAAACTCTTTTCCCACTG
TTTAAGGAGTTAGTGGATTACTGCCATTCACTTCATAATCCAGTAGGATC
CAGTGATCCTTACAAGTTAGAAAACATAATCTTCTGCCTTCTCATGATCC
AACTAATGCCTTACTCTTCTTGAAATTTTAACCTATGATATTTTCTGTGC
CTGAATATTTGTTATGTAGATAACAAGACCTCAGTGCCTTCCTGTTTTTC
ACATTTTCCTTTTCAAATAGGGTCT

>HU-03125-3 (680 bp)

GAGTTGCTGCAAACCTGACCCCTGCTCAGTAAAGCACTTGCAACCGTCTG
TTATGCTGTGACACATGGCCCCTCCCCCTGCCAGGAGCTTTGGACCTAAT
CCAAGCATCCCTTTGCCCAGAAAGAAGATGGGGGAGGAGGCAGTAATAAA
AAGATTGAAGTATTTTGCTGGAATAAGTTCAAATTCTTCTGAACTCAAAC
TGAGGAATTTCACCTGTAAACCTGAGTCGTACAGAAAGCTGCCTGGTATA
TCCAAAAGCTTTTTATTCCTCCTGCTCATATTGTGATTCTGCCTTTGGGG
ACTTTTCTTAAACCTTCAGTTATGATTTTTTTTTCATACACTTATTGGAA
CTCTGCTTGATTTTTGCCTCTTCCAGTCTTCCTGACACTTTAATTACCAA
CCTGTTACCTACTTTGACTTTTTGCATTTAAAACAGACACTGGCATGGAT
ATAGTTTTACTTTTAAACTGTGTACATAACTGAAAATGTGCTATACTGCA
TACTTTTTAAATGTAAAGATATTTTTATCTTTATATGAAGAAAATCACTT
AGGAAATGGCTTTGTGATTCAATCTGTAAACTGTGTATTCCAAGACATGT
CTGTTCTACATAGATGCTTAGTCCCTCATGCAAATCAATTACTGGTCCAA
AAGATTGCTGAAATTTTATATGCTTACTGA
